# Relationship between Oxygen Uptake Reserve and Heart Rate Reserve in Young Male Tennis Players: Implications for Physical Fitness Monitoring

**DOI:** 10.3390/ijerph192315780

**Published:** 2022-11-27

**Authors:** Jorge E. Morais, José A. Bragada

**Affiliations:** 1 Department of Sport Sciences, Instituto Politécnico de Bragança, 5300-252 Bragança, Portugal; 2 Research Center in Sports, Health and Human Development (CIDESD), University of Beira Interior, 6201-001 Covilhã, Portugal

**Keywords:** oxygen uptake, heart rate, tennis, physical fitness, training

## Abstract

The aims of this study were to (i) verify the relationship between reserve oxygen uptake (VO_reserve_) and reserve heart rate (HR_reserve_) in young male tennis players, and (ii) understand the relationship between oxygen uptake (VO_2_) measured at the end of a tennis drill and recovery heart rate (HR_recovery_) after the tennis drill. Ten young male tennis players (16.64 ± 1.69 years; 62.36 ± 6.53 kg of body mass; 175.91 ± 5.26 cm of height) were recruited from the National Tennis Association. Players were instructed to perform a tennis drill based on an incremental intensity protocol. Afterward, three levels of intensity were used based on VO_2reserve_ and HR_reserve_. A significant variance was observed between levels (VO_2reserve_ and HR_reserve_ = *p* < 0.001). VO_2reserve_ presented a significant and high agreement with HR_reserve_. The mean data revealed non-significant differences (*p* > 0.05), a very high relationship of linear regression (R^2^ = 82.4%, *p* < 0.001), and high agreement in Bland Altman plots. VO_2,_ at the highest level of intensity (>93%), presented a significant correlation with HR_recovery_ during the immediate 30 s after the drill (r_s_ = 0.468, *p* = 0.028). Tennis coaches or instructors must be aware of the differences between monitoring or prescribing training intensities based on HR_reserve_ or HR_max_. They can also use HR_recovery_ for 30 s immediately after exercise to verify and understand the variation in their players’ cardiorespiratory capacities.

## 1. Introduction

Sports modalities based on a competitive/physical activity or leisure context are often monitored to understand the effect of exercise/practice on the overall physical fitness of athletes or participants. The best way to measure the intensity of a given exercise is through oxygen uptake (VO_2_) [1]. This measures an athlete/participant’s ability to intake oxygen through the respiratory system and deliver it to all working tissues and muscles [2]. Like VO_2_, HR also increases with exercise intensity to respond to the increased metabolic demands of muscles and other tissues [2]. Thus, for convenience (based on simpler and less expensive equipment), exercise intensity is usually monitored through heart rate (HR) [3,4]. This correct and valid procedure is based on the fact that HR presents an almost perfect linear relationship with oxygen consumption [5]. Therefore, HR has been used for several decades by researchers and coaches to monitor the physical fitness status of athletes [6,7] and the physical activity of participants [8,9].

However, it has been indicated that there is a more accurate procedure to prescribe exercise intensities. This procedure is based on the difference between reserve VO_2_ (VO_2reserve_) and maximal VO_2_ (VO_2max_), i.e., reserve VO_2_ (VO_2reserve_) [10]. This is because VO_2_ and HR do not have absolute zeros and their maximum values vary according to individual intrinsic characteristics [11]. Indeed, studies have shown that VO_2reserve_ and HR_reserve_ are more closely correlated than VO_2max_ and HR_max_ [11,12]. Consequently, the use of this procedure engages a more accurate exercise prescription because it is based on each athlete/participant’s lowest and highest VO_2_ and HR values. Nevertheless, as far as is understood, it appears that this is not a standard procedure used by researchers and coaches in the tennis context. There are studies that have used VO_2reserve_ and HR_reserve_ to understand the exercise intensity or prescription [13,14], and others have used VO_2max_ or HR_max_ [15,16]. Therefore, it could be argued that researchers and coaches use one or the other without any reasoning behind the choice. Once again, it was not possible to find any information about the relationship between VO_2reserve_ and HR_reserve_ in tennis players. This study will bring deeper insights into training monitoring and prescribing.

Researchers and coaches, regardless of the sport or physical activity, are always looking for new protocols or tests that allow them to have immediate feedback on the overall physical fitness of their athletes [17]. These aim to be simple and non-invasive protocols/tests providing coaches and athletes or participants with immediate outputs. Furthermore, it was reported that recovery heart rate (HR_recovery_), i.e., recovery immediately after exercise, can be a strong indicator of the athlete’s or participant’s cardiorespiratory capacity [18,19]. Thus, measuring the decrease in HR during recovery immediately after the end of the exercise is considered a simple, valid, and non-invasive procedure for understanding cardiorespiratory fitness [20,21]. This procedure has the additional advantage of being easily applied in different situations and with commercial equipment that allows the measurement of HR. However, there is no specific information in the literature about the relationship between VO_2_ at the end of an exercise and HR_recovery_. Understanding this relationship may provide coaches and athletes/participants with a practical tool to measure their physical fitness.

Therefore, the aims of this study were to (i) verify the relationship between VO_2reserve_ and HR_reserve_ in young male tennis players and (ii) analyze the relationship between VO_2_ measured at the end of a tennis drill and HR_recovery_, i.e., after the tennis drill. It was hypothesized that a high and strong relationship would be verified between VO_2reserve_ and HR_reserve_. Moreover, players with higher VO_2_ at each intensity level would be more likely to recover more beats/min after a tennis drill. 

## 2. Materials and Methods

The sample consisted of 10 young male tennis players (16.64 ± 1.69 years; 62.36 ± 6.53 kg of body mass; 175.91 ± 5.26 cm of height) recruited from the National Tennis Association. At the time of data collection, they were ranked in the national top 50. The inclusion criteria for the participants were (i) being a national-level tennis player and (ii) not having interruptions in daily training. Parents or guardians and players signed an informed consent form. All procedures were in accordance with the Declaration of Helsinki regarding human research, and the Polytechnic Ethics Board approved the research design (Nr. 75/2022).

### 2.1. Experimental Protocol

**Figure 1:**
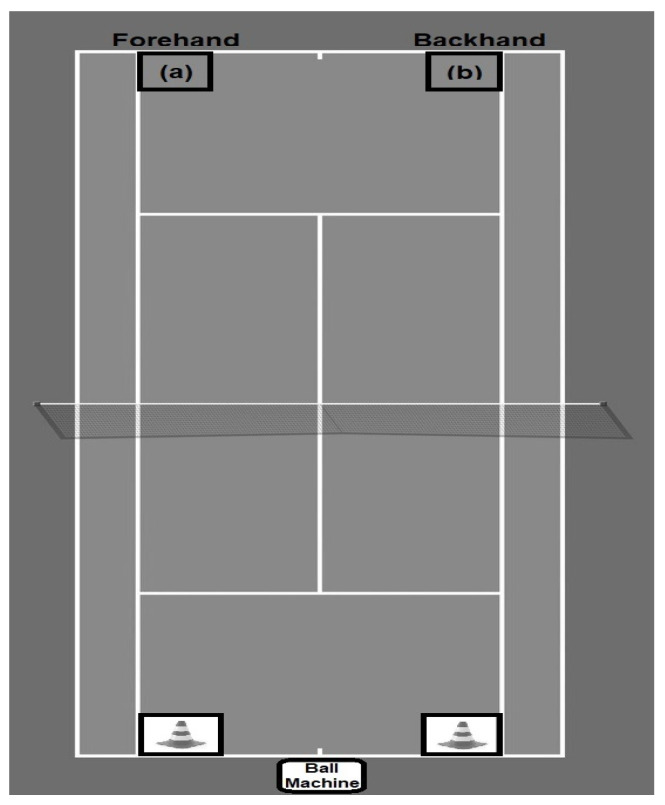
Experimental protocol. (**a**) indicates landmark for the forehand stroke; (**b**) indicates landmark for the backhand stroke; cones indicate the indicative target.

Before data collection, players performed a warm-up dedicated to tennis [22]. Afterward, they had a 5 min period to familiarize themselves with the experimental protocol. This consisted of a two-line-wide mode drill test. Players had to alternate between hitting a wide forehand and a wide backhand [13]. A ball machine (Spinfire 2 Pro, Melbourne, Australia) was used to throw the balls with constant velocity (mean: ~78 km/h), always alternating the direction of the ball in the same sequence. Whenever the ball was directed to the right and left sides of the court, players were instructed to perform a forehand and a backhand stroke, respectively. To maintain and ensure the players’ concentration and strictness during the drill, they had to hit the balls on a prominent landing mark on the court. Figure 1 shows the experimental protocol.

### 2.2. Data Collection

All players were tested on an indoor hard court and under the same conditions. An incremental test with five stages was used based on the two-line-wide mode drill test. Each stage was performed for two minutes. The throwing interval of the ball was used to control the incremental test and consequently the energy demands: (i) stage 1: 12 balls/min; (ii) stage 2: 14 balls/min; (iii) stage 3: 16 balls/min; (iv) stage 4: 18 balls/min; and (v) stage 5: 20 balls/min. After each stage, players passively recovered for 60 s. The drill test ended with the players’ voluntary exhaustion or was interrupted by the researchers if the players felt exhausted. 

Before the warm-up, HR_rest_ (beats/min) was measured for 10 min while the players were sitting in silence. For the measurement of HR_rest,_ the average values of the last minute were considered. HR was measured continuously through the entire protocol (exercise and recovery). The players’ HRs were monitored with an HR monitor (Polar H9, Kempele, Finland). VO_2_ (mL/kg/min) was measured only during the recovery time after each stage. Therefore, VO_2_ at the end of each level was estimated through backward extrapolation by individual linear regression based on the HR–VO_2_ relationship [13]. Mean records every 10 s, up to the 30 s limit, were measured and registered. VO_2rest_ was considered to be 1 MET (metabolic equivalent of task; 1 MET = 3.5 mL/kg/min) [23]. Immediately after each level of the drill, players were instructed to hold their breath until the mask was placed to measure VO_2_. Although the rest time between the stages was one minute, VO_2_ during recovery was measured for 30 s. Thus, breath-by-breath gas exchange ventilatory values were continuously recorded using the Metalyzer 3B system (Cortex Biophysik, Leipzing, Germany). Gas and volume calibration of the equipment was performed before each test according to the manufacturer’s instructions. 

All tennis players performed the protocol until exhaustion, or until they could not hit the ball under acceptable conditions. The acceptance of the effort as maximal was confirmed by the fact that (i) all players reached more than 95% of the age-predicted maximum HR considering the value obtained by the following formula: HR_max_ = 208 − 0.7 × age [24], where HR_max_ (beats/min) is the maximal heart rate and age is the participant’s chronological age (years); and (ii) all players scored a 99% fatigue in the last stage of the protocol based on the Micklewright et al. scale [25]. Thus, it can be assumed that the estimated value of VO_2_ obtained in the last stage is the VO_2max_.

Data were grouped into levels based on VO_2reserve_. Three levels of intensity were used: (i) level #1 < 80%; (ii) level #2 from 81% to 93%; and (iii) level #3 > 93% [26]. For each target percentage, the following equation was used:

VO_2reserve_ = ((maximum − rest) × target percentage) + rest (1)

in which VO_2reserve_ is the reserve oxygen uptake (mL/kg/min), maximum is the maximum value of oxygen uptake (mL/kg/min), rest is the oxygen uptake at rest (mL/kg/min), and the target percentage (%) is the percentage of reserve oxygen uptake that is intended. 

### 2.3. Statistical Analysis

The Shapiro–Wilk test was used to test normality and the Levene’s test was used to test the homoscedasticity assumption in VO_2reserve_, HR_reserve_, and HR_recovery_. The mean plus one standard deviation (SD) was used as a descriptive statistic.

One-way ANOVA (*p* < 0.05) was used to verify the variance of VO_2reserve_ and HR_reserve_ (per intensity level). The variance effect size (eta square—η^2^) was computed and interpreted as (i) without effect if 0 < η^2^ < 0.04; (ii) minimum if 0.04 < η^2^ < 0.25; (iii) moderate if 0.25 < η^2^ < 0.64; and (iv) strong if η^2^ > 0.64 [27]. To understand the agreement between VO_2reserve_ and HR_reserve,_ three procedures were used: (i) mean data comparison; (ii) linear regression; and (iii) Bland Altman plots [28]. For the mean data, the independent samples t-test (*p* < 0.05) was used. The mean difference, significance value, and 95% confidence intervals (95CI) were considered. For the linear regression, the qualitative interpretation of the relationship was defined as: (i) very weak, if R^2^ < 0.04; (ii) weak, if 0.04 ≤ R^2^ < 0.16; (iii) moderate, if 0.16 ≤ R^2^ < 0.49; (iv) high, if 0.49 ≤ R^2^ 0.80; and (v) very high, if 0.81 ≤ R^2^ < 1.0 [29]. The Bland Altman analysis included the plots of the difference and average values of VO_2reserve_ and HR_reserve_ [30]. As limits of agreement, a bias of ± 1.96 standard deviation of the difference was used. For qualitative assessment, it was considered that at least 80% of the plots were within the ± 1.96 standard deviation of the difference (95CI). The Spearman correlation coefficient was used to understand the relationship between VO_2_ at the end of each level and HR during recovery (HR_recovery_).

## 3. Results

Table 1 presents the descriptive statistics of HR_reserve_ and VO_2reserve_ by stage. It is possible to observe that, for each stage increment, both the HR_reserve_ and the VO_2reserve_ increased. This indicates that an increment in the stage increased the energy demand. 

Table 2 presents the descriptive data of VO_2max_, VO_2reserve_, HR_max_, and HR_reserve_ by intensity level. A significant variance was observed in VO_2reserve_: F = 33.51, *p* < 0.001 (all pairs were significantly different *p* < 0.05), with a moderate effect size η^2^ = 0.58. HR_reserve_ presented a similar trend: F = 68.54, *p* < 0.001 (all pairs were significantly different *p* < 0.001), with a strong effect size η^2^ = 0.74.

**Figure 2:**
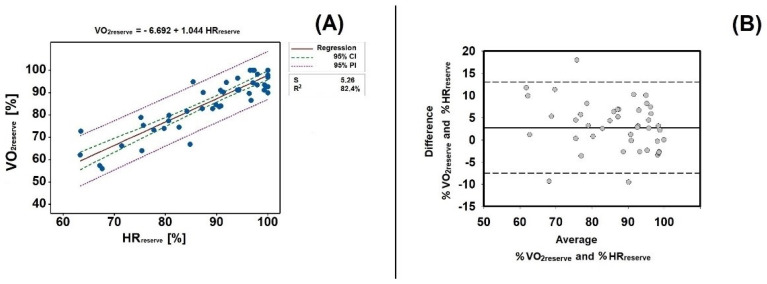
Panel (**A**)— linear regression between the percentage of VO_2reserve_ and HR_reserve_. Panel (**B**)—Bland Altman analysis between the percentage of VO_2reserve_ and HR_reserve_. VO_2reserve_—reserve oxygen uptake; HR_reserve_—reserve heart rate. 95% CI—95% confidence intervals; 95% PI—95% prediction intervals; S—standard error of estimation; R^2^—determination coefficient.

The mean data comparison revealed non-significant differences between the percentage of VO_2reserve_ and HR_reserve_ (t = 1.196, *p* = 0.234, 95CI = –1.813 to 7.321). Figure 2 presents the linear relationship between the percentage of VO_2reserve_ and HR_reserve_ (panel A), and the Bland Altman plots (panel B). A high relationship was observed (R^2^ = 82.4%, *p* < 0.001). All plots were within the 95%CI and 95%PI. As for the Bland Altman analysis, more than 80% of the plots were within the 95CI intervals. Therefore, all three criteria of agreement were met.

Table 3 presents the Spearman correlation coefficient between VO_2_ and HR_recovery_ during the first 30 s of recovery (HR_recovery (30s)_) at each intensity level. At levels #1 and #2, a non-significant correlation was found between VO_2_ and HR_recovery (30s)_. Conversely, at level #3 (highest energetic demand) a significant correlation was observed between variables. This indicates that in drills that promote greater energy demand (>93% VO_2reserve_), players who recover more beats/min in the first 30 s are more likely to present a higher VO_2_.

## 4. Discussion

The aim of this study was to verify the relationship between VO_2reserve_ and HR_reserve_ in young tennis players to understand its applicability in monitoring physical fitness and understand the relationship between VO_2_ measured during a tennis drill and recovery HR (measured immediately after the tennis drill). The main findings indicate that there is a high relationship between VO_2reserve_ and HR_reserve_ in young tennis players performing a specific tennis drill. Additionally, a significant correlation was found between VO_2_ at the end of the highest intensity level (>93%) and the corresponding HR_recovery (30s)_.

The data revealed a non-significant difference between the percentages of VO_2reserve_ and HR_reserve_ as well as a very high agreement between them. In other physical activities, such as running [11], cycling [12], or others [31], it was reported that VO_2reserve_ and HR_reserve_ present a strong relationship. Indeed, the American College of Sports Medicine [5] also recommended the use of VO_reserve_ and HR_reserve_ as the most accurate way to prescribe and monitor athletes’ or participants’ cardiorespiratory capacities. As mentioned earlier, this procedure is not always used in the tennis context. Moreover, it was not possible to find a study that verified the relationship between VO_2reserve_–HR_reserve_ in tennis players. The data showed that for young tennis players a high relationship was observed between VO_reserve_ and HR_reserve_. Tennis is a sport where performance (i.e., winning matches) may not be strictly related to cardiorespiratory capacity such as running and cycling [32,33]. Therefore, athletes or participants may present a different VO_2reserve_ or HR_reserve_ for similar performance levels. In this context, the controlling and monitoring of intensity seems more appropriate if HR_reserve_ is considered instead of HR_max_. Thus, for each participant, their individual variables, such as HR_rest_, were considered. This procedure is even more advantageous than prescribing exercise based on HR_max_, because the value is estimated. Additionally, when using estimated HR_max_, the values are the same for all participants of the same age, despite having different cardiorespiratory capacities.

Measuring HR is a simple, less time-consuming, less invasive, and cheaper alternative to using VO_2_ to measure the athletes’ or participants’ cardiorespiratory capacities. As mentioned earlier, these results indicated that VO_reserve_ and HR_reserve_ present a high relationship in young tennis players. Thus, coaches can prescribe or monitor exercise intensities based on HR_reserve_. Table 4 presents the HR_reserve_ and HR_target_ intervals for a training/practice/drill intensity based on the levels mentioned above [26]. HR_target_ is the final HR value that is provided to the athlete/participant to be achieved in training. This is displayed on the wearables commonly used by athletes/participants. Although this value is calculated accurately for the unit, it is common to indicate a range of HR values with the central value being calculated (per example: HR_target_ = 150 beats/min, ± 5 beats/min). 

Based on the data in Table 4, it is possible to observe that differences are found between the procedures, specifically between HR_target_ defined by HR_reserve_ or by HR_max_. Based on this example, it can be stated that HR_target_ is lower when prescribed by HR_max_ than by HR_reserve_, ranging between 4 and 10 beat/min. This happens because when using HR_max_, HR_rest_ is not considered. This can be a key factor for training prescription because athletes or participants with similar VO_2max_/HR_max_ can have different HR_rest_. Therefore, tennis coaches or instructors are advised to monitor the HR of their athletes or participants or prescribe exercise training intensities based on HR_reserve_ rather than on HR_max_, where the contribution of HR_rest_ is greater.

A significant and positive correlation between VO_2_ at the end of the highest level of intensity and HR_recovery (30s)_ was observed. The HR_recovery_ test is widely described as a simple and accurate procedure to assess cardiorespiratory capacity [21,34]. In fact, it has been reported that a more rapid reduction in HR immediately after exercise is associated with greater cardiovascular capacity [35]. In a review article, the main findings indicated that HR_recovery_ tends to be greater in trained participants than in untrained ones [36]. Additionally, it was suggested that for the optimal recovery values, healthy athletes can recover 60 or more beats/min during one minute [20]. These assumptions show that athletes or participants with greater cardiorespiratory capacity are more likely to present a higher HR_recovery_. Furthermore, a recent study indicated that VO_2max_ in young and healthy adults can also be predicted based on HR_recovery_ during one minute immediately after exercising [20]. These findings highlight the importance of the relationship between VO_2max_ and one-minute HR_recovery_. As mentioned before, the most common HR_recovery_ tests are based on one- or two-minute recovery, which also present a significant relationship to cardiorespiratory capacity [20,21,36]. However, the data of this study revealed a non-significant correlation between VO_2_ at the end of each level and one-minute HR_recovery_. On the other hand, it was verified that young tennis players presented a significant and positive correlation between VO_2_ at the end of the highest level of intensity (level #3: >93%) and HR_recovery (30s)_. This indicates that players or participants who presented higher VO_2_ at the end of the highest level of demand are more likely to recover more beats/min during the immediate 30 s after the drill/exercise. Therefore, it can be argued that in young tennis players, HR_recovery (30s)_ may be more related to VO_2max_ than the one-minute recovery.

Overall, these data showed that a significant and high relationship was verified between VO_2reserve_ and HR_reserve_ in young tennis players. As information is scarce about this topic in tennis, these findings may have important practical implications for monitoring and prescribing training. As shown in the given example, differences were found between using HR_reserve_ or HR_max_ for the same HR_target_. These differences were higher at submaximal levels (<93% VO_2reserve_) than at maximal or near maximal levels (>93% VO_2reserve_). Unlike the one-minute HR_recovery_, HR_recovery (30s)_ presented a significant and positive correlation to VO_2_ at the end of the highest intensity level (>93% VO_2reserve_). This indicates that, at least in young tennis players, the first 30 s immediately after exercise are more related to greater cardiorespiratory capacity. In general, the present findings indicate that coaches or instructors are advised to use HR_reserve_ to establish HR_targets_. In addition, they can also monitor their training program’s effects (in a cardiorespiratory capacity perspective) using HR_recovery (30s)_ at intensities > 93% VO_2reserve_ (i.e., HR_reserve_, as a significant and high relationship was verified between these two variables). That is, players or participants who increase their HR_recovery (30s)_ are also improving their cardiorespiratory capacity.

As the main limitations, it can be considered that: (i) a large sample size may present more consistent findings; (ii) these outputs are only suitable for young male tennis players; and (iii) the experiment was only measured once. Thus, it can be argued that the results of the experiment may have been influenced by the previous day’s sleep, weather, diet, and other factors that could also have affected the results of the physiological parameters. Therefore, future studies on this topic may consider establishing the relationship between VO_2reserve_ and HR_reserve_ in elite or recreational tennis players, as well as in female participants. Moreover, it is also important to understand whether a larger sample size or different participant demographics will present different results in HR_recovery_. In addition, applying the same experiment twice will help to verify the reliability of the outputs.

## 5. Conclusions

A significant and high relationship was observed between VO_2reserve_ and HR_reserve_ in young male tennis players. This means that HR_reserve_ can be used as a substitute for VO_2reserve_ in daily training. In addition, these findings suggest that tennis coaches and instructors must be advised about the differences of monitoring and prescribing training intensities based on HR_reserve_ or HR_max_. They are recommended to use the former for accurate results. HR_recovery (30s)_ was significantly correlated with VO_2_ at the end of the highest demanding intensity drill (>93% VO_2reserve_). So, as HR_reserve_ significantly represents VO_2reserve_, coaches and instructors could use this simple protocol to understand if their players improved their cardiorespiratory capacities immediately after exercises >93% HR_reserve_.

## Figures and Tables

**Table 1:** Descriptive statistics of HR_reserve_ and VO_2reserve_ based on the levels performed during the experimental protocol.

Machine Stage	Mean ± 1SD	
	HR_reserve_ (beats/min)	VO_2reserve_ (mL/kg/min)
1	106.3 ± 16.3	27.8 ± 5.9
2	117.7 ± 19.8	32.0 ± 8.7
3	126.5 ± 15.4	34.7 ± 7.6
4	135.1 ± 11.4	36.7 ± 6.3
5	140.3 ± 9.2	38.5 ± 5.5
Average	126.0 ± 18.6	34.2 ± 7.6

Stage—corresponds to the categorization of the test’s intensity; HR_reserve_—reserve heart rate; VO_2reserve_—reserve oxygen uptake.

**Table 2:** Values of VO_2max_, VO_2reserve_, HR_max_, and HR_reserve_ per intensity level of VO_2reserve_.

	Mean ± 1SD			
	VO_2max_ (mL/kg/min)	VO_2reserve_ (mL/kg/min)	HR_max_ (beats/min)	HR_reserve_ (beats/min)
**Level #1—VO_2reserve_ < 80%**	29.0 ± 5.6	25.5 ± 5.6	157.9 ± 13.7	102.5 ± 13.1
**Level #2—81% < VO_2reserve_ ≤ 93%**	38.1 ± 4.6	34.6 ± 4.6	186.7 ± 12.0	131.3 ± 11.0
**Level #3—VO_2reserve_ > 93%**	43.0 ± 4.8	39.5 ± 4.8	192.7 ± 11.5	137.5 ± 10.4

VO_2max_—maximal oxygen uptake; VO_2reserve_—reserve oxygen uptake; HR_max_—maximal heart rate; HR_reserve_—reserve heart rate.

**Table 3:** Spearman correlation coefficient between VO_2_ and HR_recovery (30s)_ by intensity level. It also presents the beats/min (mean ± 1 SD) recovered in each level during the immediate 30 s after the drill.

	VO_2_ Level #1	VO_2_ Level #2	VO_2_ Level #3
**HR_recovery(30s)_ level #1**	30.14 ± 9.13r_s_ = 0.343 (*p* = 0.230)		
**HR_recovery(30s)_ level #2**		26.17 ± 8.20r_s_ = −0.068 (*p* = 0.810)	
**HR_recovery(30s)_ level #3**			21.91 ± 6.42r_s_ = 0.468 (*p* = 0.028)

HR_recovery (30s)_ —recovery heart rate for 30 s; VO_2_—oxygen uptake.

**Table 4:** Training intensities based on individual HR_reserve_.

	HR_reserve_		HR_max_	Difference (beats/min)
	HR_reserve_ (beats/min)	HR_target_ (beats/min)	HR_target_ (beats/min)	
**HR_reserve_ < 80%**	<120	<170	<160	10
**HR_reserve_ (80** **%–93%)**	(120–140)	(170–190)	(160–186)	10–4
**HR_reserve_ > 93%**	>140	>190	>186	>4

HR_reserve_—reserve heart rate; HR_target_—target heart rate to be achieved for practice/training.

## Data Availability

Not applicable.
